# Enzymatic hydrolysis as a tool to improve total digestibility and techno-functional properties of pigeon pea (C*ajanus cajan*) starch

**DOI:** 10.1016/j.heliyon.2021.e07817

**Published:** 2021-08-16

**Authors:** Ricardo Benítez Benítez, Wilmar Fernando Elvira Tabares, Luis Alberto Lenis Velásquez, Clara Inés Hurtado Sánchez, Omar Alberto Salinas Cruel

**Affiliations:** Group of Natural Products Chemistry (QPN), Department of Chemistry, Universidad del Cauca, (501100005682) career 3 No. 3N-100, Popayán, Colombia

**Keywords:** Enzymatic hydrolysis, Starch, Pigeon pea, Total digestibility

## Abstract

Recent studies have indicated that starch from legumes can potentially be used as an alternative to commercial flour with applications in food and biomaterials; however, some modifications may be required first to improve their functionality, as they show relatively lower solubility and functional properties compared to commonly marketed flours (e.g. good water retention capacity). This work used multiple enzymes in flour extracts of pigeon pea (*Cajanus cajan*), a legume, to optimize the enzyme hydrolysis process of such extracts by the Response Surface Method (RSM), to increase the digestibility and obtain desirable functional attributes at the nutritional level. The pH, temperature, time and enzyme/substrate (E/S) ratio were evaluated, and the degree of hydrolysis (DH) was calculated as well as the reducing sugar content (%RS), used as response variable. According to the experimental design, the best pH, temperature, time and E/S ratio were 6.8, 43 °C, 1.84% m/m and 270 min, respectively. The %RS for the samples under optimal conditions was 3.49 ± 0.02%, and the *in vitro* digestibility yielded values of 39.2 ± 0.4, 58.6 ± 0.3 and 2.2 ± 0.2 for slowly digestible starch (SDS), rapidly digestible starch (RDS) and resistant starch (RS), respectively. Total digestibility (TD) was 97.8 ± 0.5. The statistical analysis revealed a strong positive relationship for E/S ratio followed by pH: (E/S) ratio, temperature and pH. Enzymatic hydrolysis carried out on pigeon pea showed an increase in TD. Viscosity, water retention capacity (WRC) and solubility were evaluated showing good response for future applications at the industrial level.

## 1 Introduction

**Figure 1:**
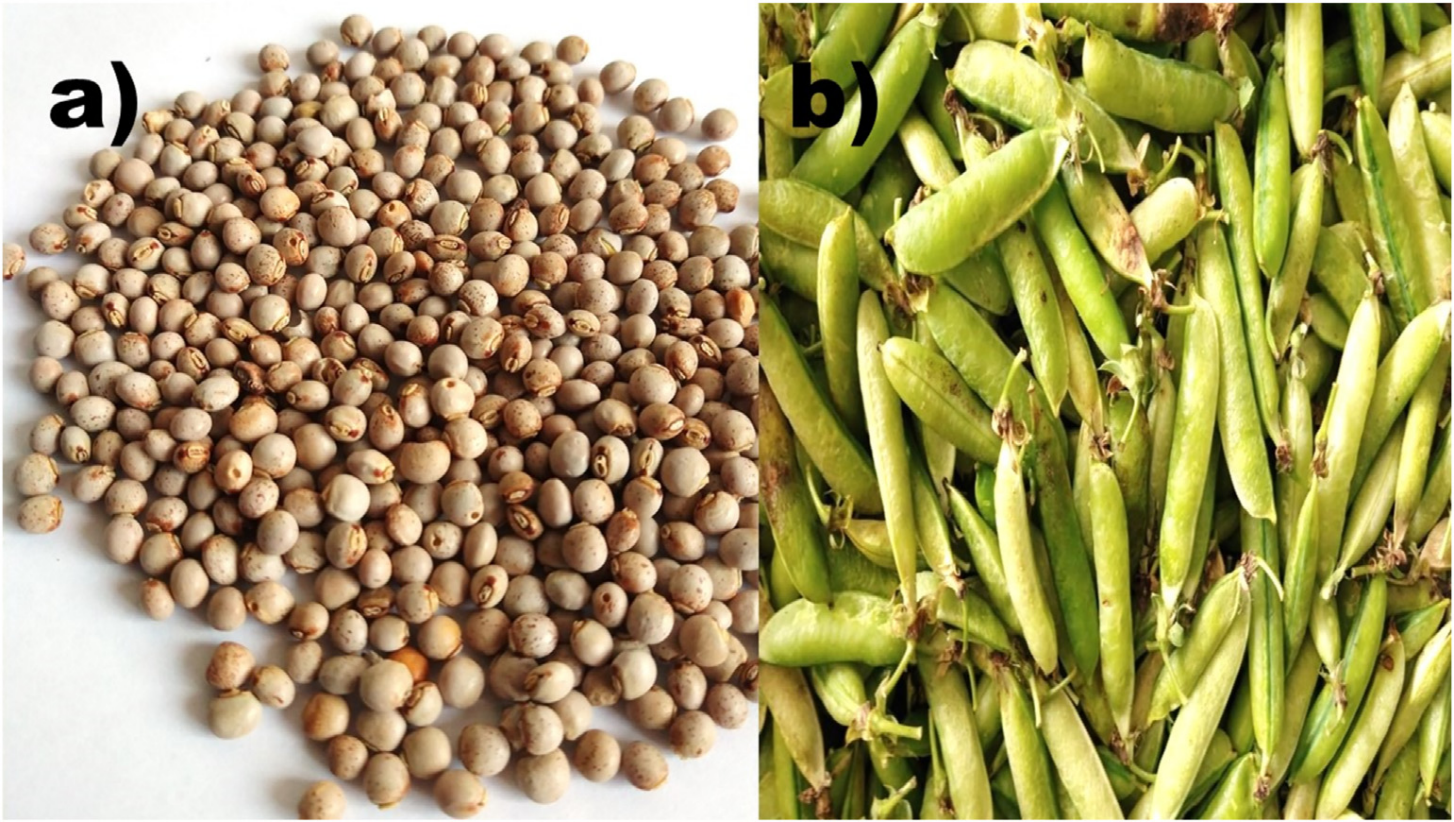
Seed (a) and pod (b) of *Cajanus cajan. Own elaboration.*

Pigeon pea (*Cajanus cajan*) is a legume with high nutritional value, easy to produce in many marginal areas where other crops are grown. This legume has been cultivated in India over 4000 years. It then spread to China and some parts of Africa (Castillo-Gómez et al., 2016) (Zhang et al., 2018). It has been used for both human and animal consumption in the form of whole debarked seeds or flour. It is also widely used in India for medicinal purposes and in Latin America at the nutritional level (Correa and Henry, 1989). In Colombia, it is grown mainly on the Atlantic Coast and in the Andean region (Castillo-Gómez et al., 2016). It can be prepared in many forms and its main consumption is as cooked grain, in soups or stew, although the nutritional and functional properties allow its use as flour cooked to obtain a wide range of products (Torres and Guerra, 2003). Legume consumption has been associated with positive health effects in various studies (Rovalino-Córdova et al., 2018). The *Cajanus cajan* legume comes in pods that contain between two and six seeds of varying color (see Figure 1). Pigeon pea is found as a round-shaped seed also known by the names of tree bean, Congo pea, Guandu, No-eye pea, Red gram, Arhur, Grandul, Dhal, Toor, Grinds pea, Puerto Rico pea, Urhur, Feijao Guandu, Adhaki, Chieh Tu Tzu, Chieh Tu, Gandul, Guaduli, Guandul, Pois Cajan, Pois Congo, Pois D'Angolie, Shan Tou Ken, Kachang gude and quinchoncho and the grain can measure approximately 6mm in length (Torres and Guerra, 2003) (Castillo-Gómez et al., 2016).

According to studies determining the chemical composition and nutritional content of the raw seeds, these are a good source of carbohydrates, followed by proteins (Navarro et al., 2014). *Cajanus cajan* has been spread into China over a 1000 year history and cultivated in the provinces of Hainan, Guangxi, Guangdong and Yunnan. The pigeon pea seed contains 57.3–58.7% carbohydrates, 1.2–8.1% crude fiber, 0.6–3.8% lipids, 18.6–22.0% crude protein and 3.7–4.9% ash (Machacon et al., 2018) (Akand et al., 2010) (Etonihu et al., 2009) (Butt and Batool, 2010). Moreover, this legume has a high starch content, a characteristic that reduces its useful life, because it is grown mainly in tropical areas with high levels of humidity. Despite being a food rich in protein and starch, it has not been properly exploited (Zhang et al., 2018).

The starch in these seeds can be processed for direct food consumption, for pharmaceutical products, and as fermentable sugar for other diverse products by means of bioconversion. The physical properties of this constituent depend on its composition, which shows variability between plants and even between species, limiting its subsequent application in technological processes and influencing its biological quality due to antinutritional factors that are generating unfavorable conditions for digestibility (Englyst et al., 1992). Pigeon pea, despite being a seed that adapts to different soil and climatic conditions and that requires little fertilization – which thereby reduces cultivation and production costs – does not enjoy widespread cultivation or use as one option among other common plant sources so that studies of this plant material are scarce compared with other legumes (Acevedo et al., 2020). Starch being a source of energy, nutrient absorption is reduced due to its poor digestibility, but improves following hydrolysis by α-amylase and glucosidase in the human small intestine. Several factors affect the hydrolysis process, prominent among which are pH, temperature, time of reaction, concentration of enzyme and substrate, type and/or pretreatment of the starch, and stirring speed. Starch is classified into 3 categories: slowly digestible starch (SDS), rapidly digestible starch (RDS) and resistant starch (RS), depending on how it is digested - slowly, quickly or resists digestion, respectively. This therefore becomes a tool with which to evaluate digestibility *in vitro*, enabling decisions to be made as to kind of products in which it can be used (Kawaljit and Seung-Taik, 2008). The aim of this research was to establish the optimal conditions for enzymatic hydrolysis in pigeon pea flour and to evaluate the effect of this process on the digestibility and techno-functional properties of the flour extracts obtained, in order to promote the widespread use of this crop as an alternative in applications at the food level.

## 2 Materials and methods

### 2.1 Materials

Commercial Amylase was used as α-amylase (EC.3.2.1.1.) produced by *Bacillus* spp. (solid state). The Amylase™ AG 300L (Gluco-amylase), Dextrozyme ® GA 1.5X (Gluco-amylase) are presented as a commercial liquid preparation, from Novozymes, with a declared activity of 300 AGU/mL and 400 AGU/g respectively and are produced by *Aspergillus niger*.

### 2.2 Preparation of pigeon pea flour

The pigeon pea samples were supplied by farmers from the village of La Carbonera, in the Municipality of Bolívar, in the Cauca Department of southwest Colombia. This crop is linked to the *Cauca Sin Hambre* Foundation (Cauca Free of Hunger) a project of the *Gobernación del Cauca*, the local governing authority.

Pigeon pea was processed into flour using the procedure by Harinder et al. (1999). The fresh grains were cleaned, washed with distilled water and dried in the sun for 8 h. They were then ground using an IMUSA® Pica 123 Plus mill to obtain a flour with homogeneous particle size when passed through a N°10 sieve. Finally, approximately 100 g samples were extracted by the quartering technique.

### 2.3 Proximal analysis of the pigeon pea seed

The proximal analysis of the crushed pigeon pea seeds was carried out using the methodology shown in Table 1.

**Table 1:** Methods used for the proximal analysis of the pigeon pea seed.

Determination	Description	Method
Moisture	Dehydration at 100–105 °C in a constant pressure oven until constant weight	AOAC 950.43
Ash	Calcination at 550 °C for 4 h	AOAC 942.15
Ethereal extract	Soxhlet ​extraction ​for 4 h	AOAC 920,153
Crude protein	Kjeldahl: Acid digestion and nitrogen distillation (N x 6.25)	AOAC 968.06 (adapted)
Crude fiber	Weende: ​Acid-base digestion and calcination	AOAC 962.09
Non-nitrogenous extract (NNE)	By difference	

### 2.4 Enzyme modification

The enzymatic modification was carried out according to the methodology modified from Bombara et al. (1997). Pigeon pea flour suspensions were prepared taking 100 g samples and bringing them to a volume of 1L in a buffer of citric acid (0.10 M) and phosphate or sodium acid (0.20 M) (pH 6.9 according to test). Subsequently, heating was carried out at 90 °C for 30 min. The enzyme complex was added after the chosen temperature for each of the experiments was reached, according to the enzyme/substrate (E/S) ratio (m/m) established (0.16, 0.50, 1.0, 1.5, 1.8). The reaction mixture was placed in a bioreactor with a capacity of 20 L (Centricol) in the Biotechnology Laboratory of the Faculty of Agricultural Sciences of the University of Cauca. The bioreactor offers automatic control of temperature and stirring speed. The ranges employed to evaluate variables pH, temperature and time were 3.5–6.0, 50–70 °C and 120–300 min, respectively. Separation between solid and supernatant was carried out for each sample once the enzymatic activity had ended, by heating at 90 °C for 20 min. After decanting the samples, the supernatant was centrifuged at 4500 rpm and then stored at a temperature of 4 °C for later testing. The solid residue was dried in warm air and likewise stored for use in other tests.

### 2.5 Characterization of extracts obtained from enzymatically modified pigeon pea flour

The %RS was measured by the increase in the content of reducing sugars in the supernatant (results in triplicate) by the dinitrosalicylic acid (DNS) method for reducing sugars using a GENESYS 6 UV-VIS spectrometer according to the methodology modified from Lv et al. (2020) (see Section 2.7). The solid residue was subjected to functional tests (water retention capacity, solubility and viscosity), light microscopy and *in-vitro* digestibility.

### 2.6 Determination of RDS, SDS and RS content

The RDS, SDS and RS contents were determined following the method of Englyst et al. (1992) with some modifications: Porcine pancreatic α-amylase enzyme (6.0 g, 8 × USP activity/g), (No. 7545, Sigma-Aldrich, St. Louis, MO) was dispersed in water (40.0 mL) by magnetic stirring for 10 min. Subsequently it was centrifuged at 15000 x g. The supernatant (32.0 mL) was mixed with the amyloglucosidase enzyme solution (2.0 mL, activity; 5,000–8,000 units/mL) (No. 9913, Sigma-Aldrich) and deionized water (3 mL). This enzyme solution was prepared fresh for each digestibility analysis. For the samples of native or hydrolyzed pigeon pea flour, 1 g was used in a test tube and 10 mL of guar gum solution (5 g/L in 0.05 M HCl) and 5 mL of 0.5 M sodium acetate solution (pH 5.2) were added. Only the blank was gelatinized to increase digestibility. Each was then transferred to a 50 mL test tube. The total starch content in the starch samples was measured according to Sandhu and Lim (2008). The content of RDS, SDS and RS was calculated using 30 min as incubation time. Classification of the starch, based on its digestibility was: RDS as the starch that was hydrolyzed within 30 min of incubation, RS as the starch not hydrolyzed within 120 min, and SDS as the starch digested during the period between 30 and 120 min.

### 2.7 Determination of degree of hydrolysis

For the quantitative determination of reducing sugars, the DNS (3, 5-dinitrosalicylic acid) method was followed. Miller's method (Lv et al., 2020) was used to prepare the DNS reagent containing 30 mM 3,5-dinitrosalicylic acid, 0.6 M NaOH, 0.055 M phenol, 0.05 M NaHSO_3_ and 0.7 M sodium potassium tartrate. 400 μL of RSE solution and 300 μL of DNS reagent were added to tubes, mixed and then heated in a boiling water bath for 10min. The reaction mixture was cooled immediately to room temperature and then mixed with 4.3 mL of water. A calibration curve was made by measuring the absorbance of glucose solutions of known concentration at 575 nm. Absorbance was measured using a UV–Vis spectrophotometer (UV2600, Shimazu, Japan) after 60min.

For each concentration, three measurements were made, and the data shown correspond to the mean of these results (Lv et al., 2020).

### 2.8 Experimental optimization design and statistical analysis

As a method for optimization, a factorial design with a fully raised response surface was employed, in which four factors were used: pH (3.5–6.0), temperature (50–70 °C), time (120–300 min) and enzyme/substrate concentration ratio (0.5–1.5% m/m) at three different levels. (-1, 0, and 1) and using the reducing sugar content (%RS) as the response variable. A total of 18 experiments were conducted, two of which were carried out at a value lower and higher than the range established in each factor. All the experiments were carried out in triplicate and the results expressed as the mean value ±standard deviation. The factorial design was completely random, using Statgraphics ® software (v. plus 5.1). The analysis of results to determine the significant terms was done using an analysis of variance test with a confidence level of 95%.

## 3 Results and discussion

The proximal analyses performed on pigeon pea seeds are reported in Table 2. These results confirm carbohydrates as the main fraction, the value being consistent with reports of between 58 and 64%. In the case of the crude fiber, a moderate content was obtained, with values between 7 and 10% (Navarro et al., 2014) offering the possibility of the starches being used more widely, since they can be used as raw material or as an additive in the food industry. However, despite the nutritional value yielded according to the proximal analysis, as a legume seed, antinutritive substances also tend to be present, such as amylase inhibitors, which affect the ability to absorb nutrients. Table 2 shows the results for percentage of reducing sugars in the supernatant, after the enzymatic hydrolysis process was carried out by means of an enzyme complex of amylases, and according to the four factors evaluated (pH, T, t, E/S) given the model used in the experimental design. According to the values obtained on comparing the blank and sample 4, it can be observed that the results are close because this sample corresponds to the assay that has the lowest E/S value for the range established in that factor. Likewise, for the E/S ratio between 1.00 and 1.84, the %RS values are between 1.5 and 3.5. The stability of the enzyme depends on the pH and may be associated with protonation or deprotonation processes depending on whether the pH values are low or high respectively. The temperature increases the kinetic energy of the molecules, favoring intermolecular collisions. The enzyme/substrate ratio is essential to obtaining a higher degree of hydrolysis. Using substrate concentrations that avoid enzymatic inhibition of amylases, the hydrolysis time has an influence on the digestibility and functional properties of the flours obtained. The values of the reducing sugar content are related to the degree of hydrolysis that was obtained on completion of the process. The action of α-amylase enzymes depends on the crystal structure exhibited by the starch granules (Hoover and Vasanthan 1994). If the results for samples 4 and 5 - differing only in the E/S ratio - are compared, an increase in the %RS by a factor of 3.25 is evidenced, which indicates that a greater quantity of product formed in the hydrolysis with a higher E/S ratio. The content of reducing sugars in the hydrolyzed samples shows a direct dependence on E/S ratio, which suggests that all these samples were susceptible to enzymatic attack at different levels by the amylase enzyme complex used, which implied the breakdown of α-(1–4) glycosidic bonds randomly in the starch and at the same time the release of α-dextrins (Deepika and Tulasi, 2016).

**Table 2:** Proximal analysis of pigeon pea seeds.

Determination	Result % BS ​± ​SD
Ash	3.96 ± 0.057
Ethereal extract	3.87 ± 0.071
Crude protein	22.63 ± 0.197
Crude fiber	6.53 ± 0.186
Non-nitrogenous extract (NNE)	63.02 ± 0.132

Table 3 presents ​the results for the extreme values of the factors used in the experimental design (a total 18 experiments were performed) where T is temperature, t is time and E/S is the enzyme/substrate ratio.

**Table 3:** Experiments for optimization of enzymatic hydrolysis.

Sample	pH	T (°C)	t (min)	E/S (%m/m)	% RS
Blank	6.0	70	300	-	0.52 ± 0.03 ​0
1	2.6	60	210	1.00	2.60 ± 0.14 ​0
2	4.8	43	210	1.00	2.42 ± 0.004
3	4.8	60	59	1.00	1.81 ± 0.04 ​0
4	4.8	60	210	0.16	0.62 ± 0.03 ​0
5	4.8	60	210	1.84	2.02 ± 0.03 ​0
6	4.8	60	361	1.00	1.53 ± 0.02 ​0
7	4.8	77	210	1.00	1.57 ± 0.03 ​0
8	6.8	60	210	1.00	1.77 ± 0.08 ​0
9	6.8	43	270	1.84	3.49 ± 0.02 ​0

The experimental design made it possible to ​identify and optimize ​the working limits ​to maximize the response variable (reducing sugar content, %RS). Table 4 ​shows ​the range with ​the ​minimum and maximum ​values ​in each factor and the optimal conditions in each case. ​According to ​the RSM, the optimal values for pH and E/S ratio correspond to the maximum value while for the temperature it resulted in the minimum value. ​In contrast, time ​is ​not ​located at an ​extreme ​value. ​As the enzyme used was a complex ​one (three types of amylases), ​a pH close to neutrality ​could be associated with the fact that the binding of the substrate and the release of the product from the pH in the medium are independent. ​For temperature, ​the origin of ​two of ​the amylases used ​was ​*Aspergillus ​niger;* studies from Wang et al. (2016) showed ​a maximum activity at 45 °C, a ​value very close ​to the optimal result. ​The amylase produced by the *Bacillus* ​spp. ​has ​a ​suitable ​temperature range between 40 and 50 °C ​(Ajayi and Fagade, 2007) ​for the E/S.

**Table 4:** Optimal value for enzymatic hydrolysis.

Factor	Predicted value		Optimal value
	Low	High	
pH	2.6	**6.8**	**6.8**
Temperature (°C)	**43**	77	**43**
Time (minutes)	59	361	270
E/S ratio (%m/m)	0. ​16	**1. ​84**	**1.84**

The bold correspond to the value with the higher significance.

Table 5 shows the analysis of variance of the experimental design. Here, an adequate relationship was found between the response (content of reducing sugars) and three of the four significant variables (E/S ratio, temperature and pH). It is observed that the variable that most affects the degree of hydrolysis is E/S ratio (p < 0.05, marked with a double asterisk), followed by the interaction between the pH: E/S ratio variables, and then finally those of temperature and pH (all marked with a single asterisk). All other interaction combinations and the remaining variable, time, either showed only a minimal contribution or were found to have an indirect relationship to the response variable.

**Table 5:** Level of significance (p value) of the four factors and interactions on% AR.

Factor	p value
Temperature	0.0198 ∗
pH	0.0234 ∗
Time	0.6547
E/S ratio	**0.0049** ∗∗
Temperature - pH	0.1716
Temperature - time	0.4412
Temperature - E/S ​ratio	0.2003
pH - time	0.1181
pH - E/S ratio	0.0121 ∗
Time - ​E/S ​ratio	0.3322

∗Factors with p value below 0,05. ∗∗ Factor with the greatest significance. ​No ​significant ​difference ​(p-value ​› ​0.05). The bold correspond to the value with the higher significance.

Table 6 shows the RDS, SDS and RS that was determined in native and hydrolyzed pigeon pea flour. In the reports it is found that the structure of the food is a main determinant in the speed of starch digestion. Access by digestive enzymes is difficult due to this physical structure, so it can be expected that if it is subjected to a process that modifies its structure and composition, this will generate a significant effect on starch digestibility (Wong and O'Dea, 1983) (Ogawa et al., 2018) (Hoebler et al., 1998). Native flour shows the lowest total digestibility value. The TD, (calculated from the sum of RDS + SDS) in the hydrolyzed samples increased between 19 and 31% compared to native flour, demonstrating the advantages of the enzymatic process, since various properties of legumes affect starch digestibility, including high content of viscous soluble dietary fiber constituents, the presence of various antinutrients including polyphenols and phytic acid, and the relatively high amylose/amylopectin ratio. The effect of starch structure on digestibility can also be mentioned.

**Table 6:** *In vitro* digestibility in native flour and hydrolyzed pigeon pea.

Sample	Concentration (% m/m)			
	RDS	SDS	RS	TD
Blank	29.9 ± 0.3	49.8 ± 0.4	20.3 ± 0.6	79.7 ± 0.5
Native	27.6 ± 0.8	47.3 ± 0.3	25.1 ± 0.5	74.9 ± 0.9
2	35.2 ± 0.8	59.2 ± 0.4	5.6 ± 0.4	94.4 ± 0.9
3	35.1 ± 0.9	57.5 ± 0.2	7.4 ± 0.2	92.6 ± 0.9
4	33.6 ± 0.9	55.6 ± 0.3	10.8 ± 0.3	89.2 ± 0.9
5	34.3 ± 1.2	58.8 ± 0.2	6.9 ± 0.2	93.1 ± 1.2
9∗	39.2 ± 0.4	58.6 ± 0.3	2.2 ± 0.2	97.8 ± 0.5

∗Sample 9 corresponds to the optimal sample for enzymatic hydrolysis.

Some important internal factors that affect enzymatic hydrolysis have been the subject of research for many years. Due to the complexity of starch when glucose units are organized into higher structural levels that differ in length and are formed into granules, currently no model enjoys universal acceptance. Therefore, a polysaccharide multiscale structure was used to analyze the change in starch structure during enzymatic hydrolysis. The structure of the first level consists of intact granules, depending on the source of polysaccharides (Li et al., 2020).

At the next level, the structure contains semi-crystalline growth rings consisting of alternating crystalline and amorphous lamellae. The thickness of the amorphous growth rings appears to be thinner than that of the semi-crystalline growth rings.

The third level structure consists of super double helices or a cis-trans chain structure. Super double helices are believed to be made up of interlocking chains with a linear length of many glucose units. These are packed together to form the crystallite region. The branch points of the chain correspond to the amorphous region. As individual molecules in the granule, it is assumed that the winding chain structure molecules are randomly distributed in the crystalline and amorphous regions and interspersed in close proximity to each other. Crystallization or double helices can form in the same group of branches or between adjacent groups in three dimensions, which is called a superhelical structure (Zhang. et al., 2018).

The resistant starch fraction was considerably reduced, between 57 and 91% for the hydrolyzed flours, noting that the sample carried out under optimal conditions was the one that exhibited the lowest RS value. The values obtained for RDS (27.6–39.2% m/m) and SDS (47.3–58.6% m/m), both for native and hydrolyzed flour, are higher than those obtained in another study in India, from pigeon pea starch suspensions that did not undergo enzymatic modification and whose *in vitro* digestibility was determined using porcine pancratic α-amylase (RDS = 4.2 ± 0.3%, SDS = 16.9 ± 0.7%). As a consequence of the previous values, the RS fraction in the present study is between 20.3 - 2.2% m/m, the sample with optimized hydrolysis conditions being the one that has the lowest value, and when comparing with the other study the reported value is 78.9 ± 0.8%. Although the differences in starch digestibility between species, and even in the same species, is attributed to the interaction of many factors such as granule size, amylose/amylopectin ratio, degree of crystallinity and type of crystalline polymorphic forms, the type of modification made to the material will affect the physical and structural properties and with it the biochemical characteristics (Sandhu and Lim, 2008). The blank sample that has no addition of the enzymatic complex and whose pH, temperature and time values do not correspond to the optimal ones shows a closeness with the native flour in the values obtained for *in vitro* digestibility.

Table 7 shows the results for some techno-funtional properties such as water retention capacity, solubility, gelatinization and viscosity, which allow evaluation of the changes brought about by the modification made.

**Table 7:** Techno-functional properties in native and hydrolyzed pigeon pea flour.

Sample	WRC (90°C)	Solubility (50°C)	Max. viscosity (T_g_)
Blank	6.57 ± 0.17	24.94 ± 0.004	31.1 ​(79.0)
Native	7.93 ± 0.05	24.84 ± 0.04 ​0	70.6 ​(75.6)
4	9.56 ± 0.05	24.86 ± 0.10 ​0	23.6 ​(76.7)
5	7.03 ± 0.06	24.79 ± 0.005	20.2 ​(78.2)
9	9.91 ± 0.10	24.77 ± 0.10 ​0	56.6 ​(71.6)

WRC (water retention capacity, g gel/g sample, d. b.); solubility (g soluble/g sample, d.b.); viscosity (cP, centiPoise) and gelatinization temperature, T_g_ (°C).

One of the determining factors in these techno-functional properties is the amylose content in the starch granules. WRC was evaluated at 60 °C (data not shown) and 90 °C. When the starch is heated in excess of water, an absorption and swelling process occurs. The results are in line with expectations where WRC increases with increasing temperature. The values shown in Table 5 are between 6.57 and 9.91, but the gelatinization temperatures differ. When analyzing the data, those samples with higher values of T_g_ can be associated with the fraction of amylose present in a more compact way inside amorphous regions, which restricts the swelling of the granules, such that a higher energy contribution is needed to begin gelatinization (Romero and Zhang, 2019). In hydrolyzed samples, a process of amylose degradation can be considered when comparing them with the native and the blank, which would facilitate the absorption of water by the amylopectin and the solubility of the starch.

The viscosity is associated with the particular swelling power of the chemical composition, the amylose/amylopectin ratio, the content of reducing sugars due to their constituents and the lipid and phosphorus content (Montañez et al., 2012). From the values obtained for all of the samples used in this study, the native flour is the one that shows the maximum value of 70.6 cP, which could be explained by not having suffered enzymatic action on the amylose and amylopectin molecules that make up the starch, which prevents damage to the branch points that allow swelling in the granules. For viscosity, the integrity of the amylopectin molecules exerts a stronger favorable effect. The differences observed in the hydrolyzed samples reflect a higher viscosity value in the optimized sample (S9) with 56.6 cP, followed by 23.6 and 20.0 cP for the S4 and S5 samples, respectively. Reviewing the gelatinization temperatures, S9 has the lowest T _g_ while S4 and S5 have higher T _g_ values that may be promoting a breakdown in the starch granules after swelling, which would increase the dilution of the starch. For the control sample, the viscosity value was 31.1 cP higher than S4 and S5 with a temperature close to that of these. In this case there are two factors that affect the viscosity value, the first, a high temperature that could contribute to the brittleness of the starch granules, and the second, the non-degradation of the amylose and amylopectin molecules. The viscosity value would show that the effect of temperature was partially offset by not having undergone the hydrolysis process (Miranda-Villa et al., 2013).

**Figure 2:**
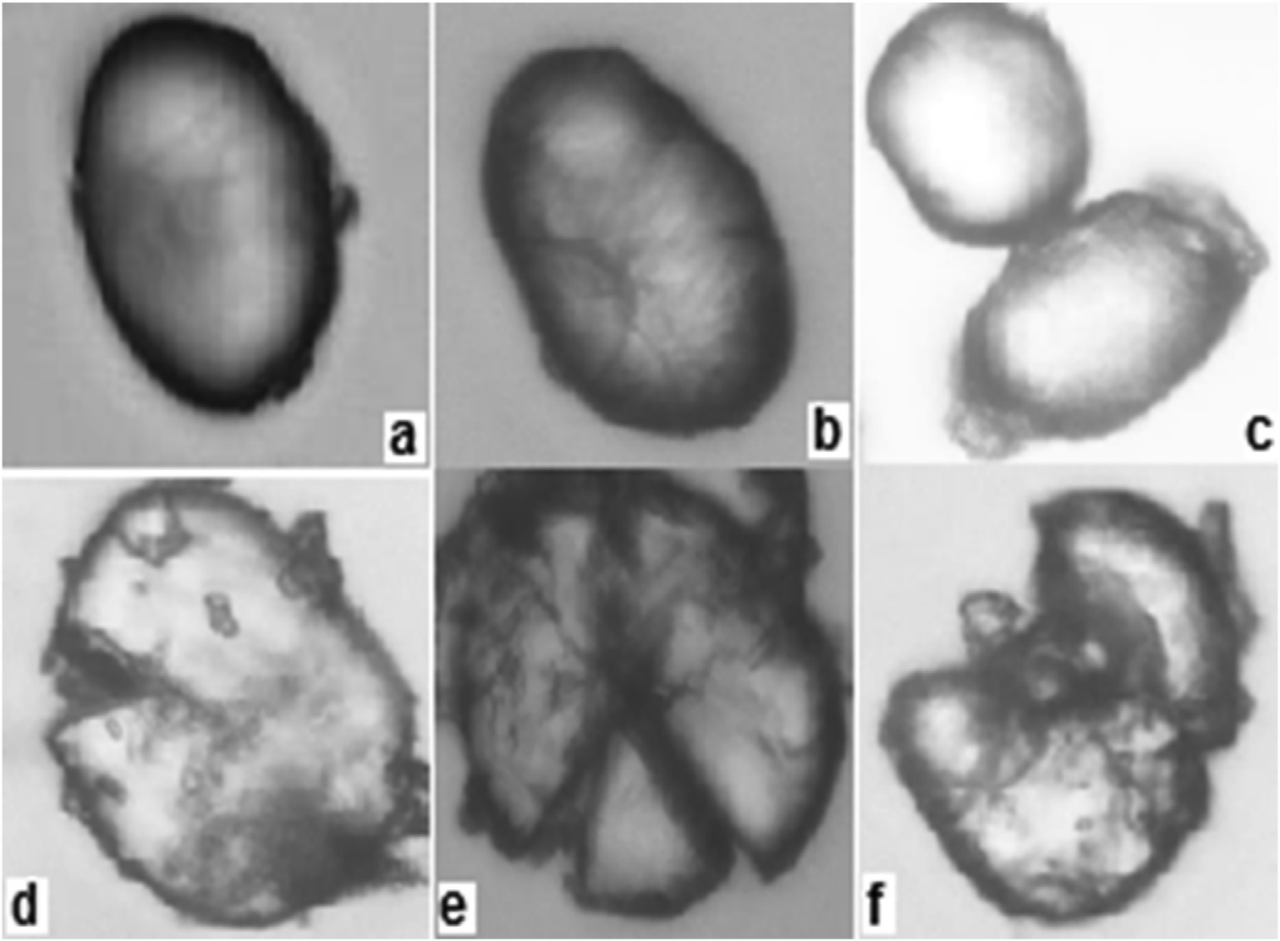
40 x (a), 100 x (b–f) microphotographs of pigeon pea flour (a: native, b: blank, c: S8, d: S2, e: S5, f: S9).

Figure 2 shows the microphotographs of pigeon pea flour, in which starch granules -corresponding to native, blank state and subsequent to enzymatic processing - are observed. The starch granules can have different shapes, among which are spherical, polyhedron, oval, bean-shaped or irregular. Generally, spherical granules can be associated with soft endosperms, unlike hard endosperms that have a polyhedral shape (Uthumporna et al., 2010). For the native and blank flours (a and b) the external surface of the granule is homogeneous, without any damage, in contrast to the hydrolyzed flours (d, e and f), in which the uniformity of the exterior part of the granules is lost, indicating that hydrolysis has occurred with regions most susceptible to this process, probably associated with the structural characteristics of starch. Likewise, in the granules of S8 (c) an intense deterioration and corrosion is not observed in the evaluated conditions, in comparison with the other hydrolyzed samples shown, S2, S5 and S9. In the S9 (optimized) sample, greater damage to the granule is noticed, which could be related to a longer hydrolysis time than in S2, S5 and S8. Enzymes manage to penetrate a food by means of diffusion on passing through the intact cell wall, making the process slow (Mishra et al., 2012); on observing the micrograph for S9 (f) it can be said that the enzymatic attack does not seem to have been limited to the surface so it is possible that enzymes have penetrated, altering the interior part of the granules (Uthumporna et al., 2010).

## 4 Conclusions

In the study carried out, differences were observed in the behavior of native flours and those subjected to enzymatic hydrolysis with α-amylases. The native starch shows lower digestibility values. Enzymatic hydrolysis allows an increase in the TD of all samples, highlighting the hydrolysis at optimal conditions (by RSM) (pH 6.8, 43 °C, E/S ratio 1.84 and 270 min) that would allow the use of this modified raw material by population groups with malnutrition problems, due to the advantages offered by the cultivation of this legume and due to its chemical composition.

Statistical analysis revealed E/S ratio as the most determining factor in enzymatic modification, followed by the interaction of pH: E/S ratio, and then the temperature and pH variables. Likewise, the results obtained in viscosity, water retention capacity, solubility and gelatinization temperature provide fundamental information to establish the appropriate conditions for technological processes that allow diversifying the uses of this legume, thus exploiting its techno-functional properties.

## Declarations

### Author contribution statement

Ricardo Benítez Benítez: Conceived and designed the experiments; Analyzed and interpreted the data; Contributed reagents, materials, analysis tools or data; Wrote the paper.

Wilmar Fernando Elvira Tabares: Conceived and designed the experiments; Performed the experiments; Analyzed and interpreted the data; Wrote the paper.

Luis Alberto Lenis Velásquez: Analyzed and interpreted the data; Contributed reagents, materials, analysis tools or data; Wrote the paper.

Clara Inés Hurtado Sánchez: Conceived and designed the experiments; Analyzed and interpreted the data; Wrote the paper.

Omar Alberto Salinas Cruel: Analyzed and interpreted the data; Wrote the paper.

### Funding statement

This research did not receive any specific grant from funding agencies in the public, commercial, or not-for-profit sectors.

### Data availability statement

Data included in article/supplementary material/referenced in article.

### Declaration of interests statement

The authors declare no conflict of interest.

### Additional information

No additional information is available for this paper.
